# Tribocatalytic Removal of Anionic and Cationic Dyes Using Pristine and Gd-, Dy-, and Er-Modified ZnO Nanorods

**DOI:** 10.3390/nano16211352

**Published:** 2026-10-24

**Authors:** Bozhidar I. Stefanov, Lyudmila Krasteva, Stanislava Todorova, Iliyana Yordanova, Simona Delibaltova, Hristo Kolev

**Affiliations:** 1 Department of Chemistry, Faculty of Electronic Engineering and Technology, Technical University of Sofia, 1756 Sofia, Bulgaria; 2 Department of Energy and Mechanical Engineering, Technical College—Sofia, Technical University of Sofia, 1756 Sofia, Bulgaria; l.krsteva@tu-sofia.bg; 3 Department of Applied Inorganic Chemistry, Faculty of Chemistry and Pharmacy, University of Sofia, 1 James Bourchier Blvd., 1164 Sofia, Bulgaria; nhtst@chem.uni-sofia.bg; 4 Institute of Catalysis, Bulgarian Academy of Sciences, “Acad. G. Bonchev” Str., Bl. 11, 1113 Sofia, Bulgaria; yordanova@ic.bas.bg (I.Y.); delibaltova@ic.bas.bg (S.D.); hgkolev@ic.bas.bg (H.K.)

**Keywords:** tribocatalysis, zinc oxide, rare-earth modification, Reactive Black 5, Brilliant Green, dye removal, mechanical energy harvesting, wastewater treatment

## Abstract

Tribocatalysis is an emerging approach for the oxidation of organic pollutants using frictional energy. ZnO nanorods were prepared by thermal decomposition of zinc acetate dihydrate and wet-chemically modified with Gd_2_O_3_, Dy_2_O_3_, or Er_2_O_3_. SEM, nitrogen sorption, XRD, ATR-FTIR, Raman spectroscopy, and XPS confirmed the formation of predominantly rod-like ZnO, preservation of its morphology and wurtzite structure after modification, and introduction of nanocrystalline RE_2_O_3_ phases. Tribocatalytic activity was evaluated under mechanical stirring using the anionic dye Reactive Black 5 (RB5) and the cationic dye Brilliant Green (BG), examining the effects of RE modification, stirring rate, friction pair, scavengers, and catalyst reuse. Removal increased with stirring rate and was consistently higher for RB5. At 800 rpm, Er:ZnO exhibited the highest activity toward both dyes, reaching approximately 81% RB5 removal (k_app_ ≈ 0.074 h^−1^) and 56% BG removal (k_app_ ≈ 0.040 h^−1^) after 24 h. COD removal reached 53–60% for RB5 and 27–43% for BG, indicating a reduction in the oxidizable organic load consistent with partial mineralization. ATR-FTIR revealed carbonate formation and PTFE transfer to recovered catalysts, highlighting surface changes and secondary contamination. The results demonstrate measurable tribocatalytic activity and show that the benefits of RE modification depend on the target dye.

## 1. Introduction

Advanced oxidation processes (AOPs) have been widely investigated for the removal of persistent organic contaminants from water. AOPs rely on the generation of highly reactive species, such as hydroxyl (•OH) and superoxide (O_2_^•−^) radicals, which can oxidize organic molecules and, under favorable conditions, lead to their complete mineralization [1,2,3,4]. In heterogeneous photocatalysis, the catalyst is a semiconductor material, which absorb light energy to generate electron–hole pairs that may either react directly with adsorbed species or participate in the formation of reactive oxygen species (ROS). Metal oxides such as TiO_2_, ZnO, WO_3_, and BiVO_4_ have therefore been studied extensively as photocatalysts for environmental remediation [1,5,6]. The efficiency of photocatalytic processes, however, may be limited by poor light penetration and non-uniform irradiation in colored, turbid, or otherwise optically dense reaction media [7,8]. This has stimulated interest in catalytic systems utilizing other forms of energy, including ferrocatalysts, in which polarization switching is induced by a fluctuating electric field [9], and pyrocatalysts, which employ cyclic temperature changes [10]. These approaches require an applied electric field or repeated thermal cycling, respectively, with the latter additionally constrained by heat-transfer rates. Neither constraint applies to mechanically driven AOPs such as piezocatalysis and tribocatalysis [4,11,12].

In piezocatalysis, deformation of a non-centrosymmetric material generates a piezoelectric potential that promotes charge separation and surface redox reactions [9,11,13]. Piezocatalytic experiments nevertheless commonly rely on high-frequency ultrasonic excitation, often at 40–60 kHz, to produce repeated deformation of the catalyst [4,11,13]. Tribocatalysis, in contrast, can utilize much lower-frequency frictional motion, commonly generated in aqueous suspensions by magnetic stirring at 200–1000 rpm (nominally 3–17 Hz) [12,14]. The current development of tribocatalysis for aqueous pollutant removal began with the work of Li et al., who reported 99% removal of Rhodamine B after 3 h from a 30 mL solution with an initial dye concentration of 5 mg L^−1^, using Ba_0.75_Sr_0.25_TiO_3_ nanoparticles at a catalyst loading of 1 mg mL^−1^ and four PTFE stirring rods operated simultaneously at 300 rpm in the dark [15].

This work stimulated growing interest in tribocatalysis; however, its fundamental mechanism remains under debate. In stirred suspensions, contact among the catalyst particles, PTFE stirring elements, and glass reactor surface is commonly proposed to generate charge through contact electrification [15,16,17]. Friction at dynamic semiconductor interfaces may additionally generate carriers through tribovoltaic processes, while alternative or complementary explanations include electronic excitation, defect generation, and localized thermal effects [11,12,18,19,20]. The generated charges may react directly with adsorbed molecules or with water and dissolved oxygen to form ROS [12,17,21,22]. Tribocatalytic performance therefore depends on the catalyst’s electronic, morphological, and surface properties, as well as experimental parameters such as the friction pair, applied load, contact frequency, and stirring configuration [18,22].

Among the metal oxides investigated for mechanically driven catalysis, ZnO represents a promising tribocatalytic platform because of its low cost and availability, semiconducting properties, and non-centrosymmetric wurtzite structure [11,16,17,21,23]. Its tribocatalytic activity was first demonstrated using hydrothermally prepared ZnO nanorods, which achieved approximately 99.8% Rhodamine B removal after 60 h (5 mg L^−1^ Rhodamine B, 50 mL; 1 mg mL^−1^ catalyst; 1000 rpm) [16]. Subsequent comparative studies confirmed a strong dependence on ZnO morphology and structure but reached different conclusions regarding the governing material characteristic. For hydrothermally prepared nanowires, nanoclusters, and nanostars, Rhodamine B removal increased from 80% to 95% with increasing specific surface area (5 mg L^−1^ Rhodamine B, 50 mL; 2 mg mL^−1^ catalyst; 12 h) [17]. In contrast, highly crystalline prismatic particles outperformed smaller agglomerated nanoparticles despite their lower specific surface area, with the apparent rate constant increasing from approximately 0.031 to 0.066 min^−1^ (5 mg L^−1^ Rhodamine B, 30 mL; 10 mg mL^−1^ catalyst; 400 rpm; 90 min) [23]. Together, these findings indicate that crystallinity and the capacity to sustain frictional forces may be more influential than specific surface area in ZnO tribocatalysis. Effective use of coarser particles could also facilitate catalyst separation and recovery after treatment.

Strategies established in photocatalysis, including defect and heterojunction engineering, are increasingly being applied to tribocatalysts to promote the separation and transfer of friction-induced charges [12,22]. Rare-earth (RE) modification has similarly been used to alter the electronic structure and charge-carrier dynamics of ZnO, with Gd-, La-, Nd-, Sm-, Dy-, and Er-modified materials exhibiting modifier-dependent optical and photocatalytic properties [24,25,26,27]. More specifically, enhanced photocatalytic dye removal has been reported for Gd-modified ZnO nanoparticles, Dy-modified ZnO nanoparticles, and Er-modified ZnO nanorods prepared by thermal decomposition [28,29,30]. Nevertheless, the effects of Gd, Dy, and Er modification on the tribocatalytic behavior of ZnO have not been systematically compared.

Organic dyes are frequently employed to screen tribocatalytic materials. However, tribocatalytic performance determined using a single dye may not be representative of other contaminants because dye charge and functional groups, together with catalyst surface state and solution pH, govern adsorption and may alter the relative contributions of surface-mediated charge transfer and liquid-phase ROS reactions [31,32]. Although Rhodamine B, Methyl Orange, Methyl Violet, and Methylene Blue dyes have been used in previous tribocatalytic studies [15,16,32], the influence of dye charge on the apparent activity of ZnO tribocatalysts has received comparatively little attention. Comparing oppositely charged contaminants can therefore provide a more discriminating assessment than screening with a single dye.

Accordingly, the present work addresses two related questions: whether surface modification with Gd, Dy, or Er alters the tribocatalytic behavior of ZnO nanorods and whether the resulting activity trends remain consistent for oppositely charged contaminants. Pristine and RE-modified ZnO nanorods obtained through a simple thermal-decomposition and incipient-wetness route were therefore compared using the anionic azo dye Reactive Black 5 and the cationic triarylmethane dye Brilliant Green.

## 2. Materials and Methods

### 2.1. Synthesis and Rare-Earth Modification of ZnO Nanorods

**Figure 1:**
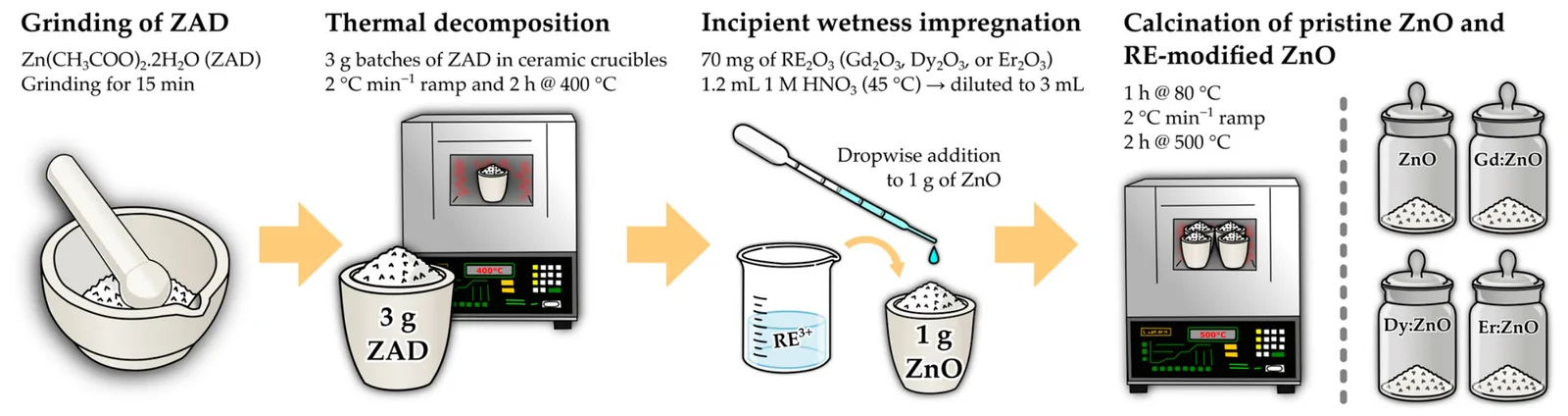
Schematic illustration of the preparation of pristine and rare-earth-modified ZnO tribocatalysts (RE = Gd, Dy, or Er).

The preparation procedure is schematically illustrated in Figure 1. Pristine ZnO was synthesized by thermal decomposition of zinc acetate dihydrate (ZAD; Zn(CH_3_COO)_2_.2H_2_O, ACS grade, ≥98.0%, Thermo Scientific Chemicals, Waltham, MA, USA). After grinding for 15 min, the precursor was divided into 3 g batches in loosely covered ceramic crucibles and heated to 400 °C at 2 °C min^−1^, with a dwell time of 2 h. The resulting ZnO powder was reground and divided into 1 g batches.

Rare-earth modification was performed by incipient wetness impregnation. Gd_2_O_3_, Dy_2_O_3_, or Er_2_O_3_ (70 mg; REacton^®^, 99.99% REO, Thermo Scientific Chemicals, Waltham, MA, USA) was dissolved separately in 1.2 mL of 1 M HNO_3_ at 45 °C, and each solution was diluted to 3 mL with deionized water. The resulting nitrate solutions were added dropwise to separate 1 g ZnO batches to obtain a nominal RE content of approximately 3 at. %, followed by thorough homogenization. The impregnated powders and an unmodified ZnO batch were maintained at 80 °C for 1 h, heated to 500 °C at 2 °C min^−1^, and held for 2 h. After cooling, the powders were reground and used without further treatment. The final materials are hereafter denoted as ZnO, Gd:ZnO, Dy:ZnO, and Er:ZnO.

### 2.2. Characterization Techniques

SEM imaging and EDS analysis were performed using a Zeiss EVO 15 microscope (Carl Zeiss Microscopy GmbH, Oberkochen, Germany) equipped with an AZtecLive One E detector (Oxford Instruments, Abingdon, UK). The SEM was operated at an accelerating voltage of 30 kV and a working distance of 8 mm.

Nitrogen sorption measurements were performed at 77.4 K using a NOVA 1200e analyzer (Quantachrome Instruments, Boynton Beach, FL, USA). Before analysis, the samples were degassed at 150 °C for 18 h. Specific surface areas were calculated using the Brunauer–Emmett–Teller (BET) method.

XRD patterns were recorded over the 2θ range of 10–90°, with an angular increment of 0.029°, using a Bruker D8 Advance diffractometer (Bruker AXS GmbH, Karlsruhe, Germany) with Cu Kα radiation and a LynxEye detector. Instrumental broadening, determined using a corundum standard, was 0.02° in 2θ. Rietveld refinement was performed using PowderCell software (version 2.3).

Raman spectra were collected using a TO-ERS-532 spectrometer (Thunder Optics S.A.S., Montpellier, France), a 532 nm laser operated at 30 mW, and a Raman probe with a 20× microscope objective. Each spectrum represents the average of 10 acquisitions with an integration time of 500 ms. Attenuated total reflectance Fourier-transform infrared (ATR-FTIR) spectra were recorded using a Cary 630 spectrometer equipped with a diamond ATR accessory (Agilent Technologies Inc., Santa Clara, CA, USA).

XPS measurements were performed using an ESCALAB MkII electron spectrometer (VG Scientific) with non-monochromatic Al Kα radiation (hν = 1486.6 eV). The pressure during analysis was approximately 4 × 10^−9^ mbar. Pass energies of 20 eV and 50 eV were used for the Zn 2p/O 1s and RE core-level regions, respectively. The instrumental energy resolution was approximately 1 eV, based on the Ag 3d_5/2_ peak. The binding-energy scale was referenced to the Zn 2p peak at 1021.7 eV. Spectra were processed using SpecsLab2 and CasaXPS (version 2.3.26PR1) by subtracting the X-ray satellite contributions, applying a Shirley-type background [33], and fitting symmetric Gaussian–Lorentzian line shapes. Elemental concentrations were determined from the fitted peak areas using Scofield photoionization cross-sections [34].

### 2.3. Determination of Tribocatalytic Activity

**Figure 2:**
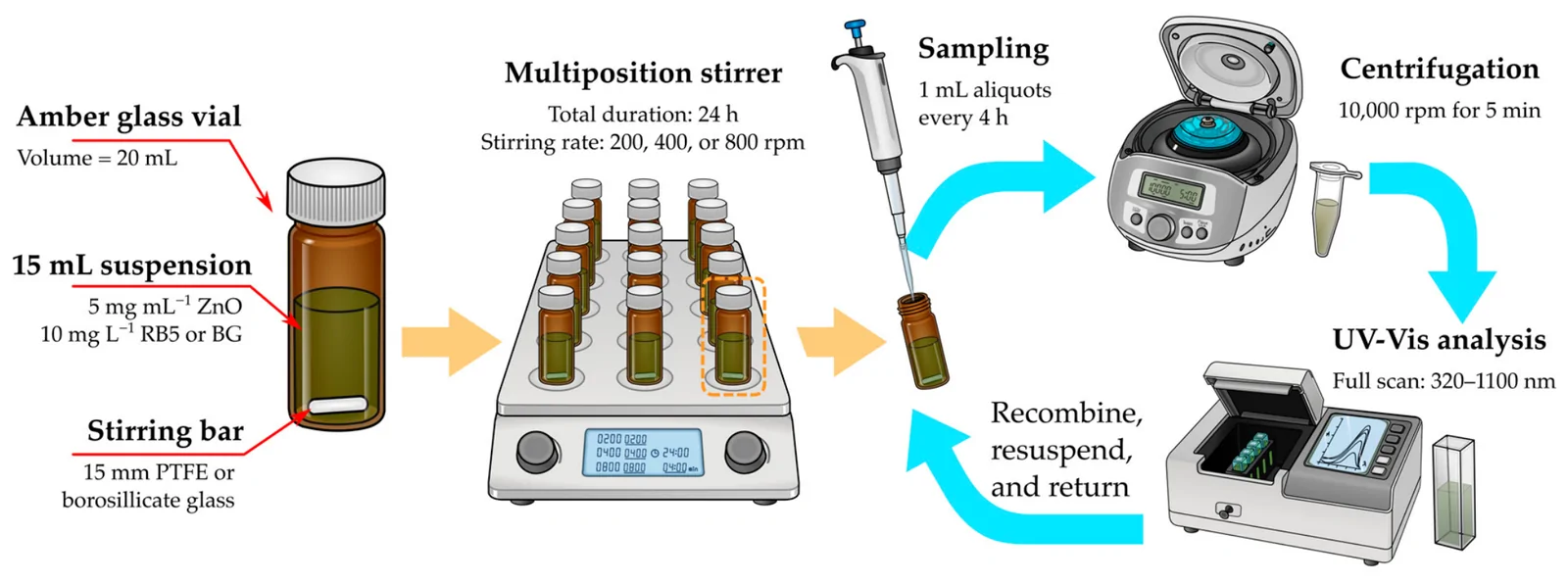
Schematic illustration of the experimental procedure used to evaluate the tribocatalytic activity of the ZnO-based catalysts.

The tribocatalytic activity of pristine and RE-modified ZnO was evaluated using the anionic dye Reactive Black 5 (RB5) and the cationic dye Brilliant Green (BG), both of technical grade (Sigma-Aldrich, Merck KGaA, Darmstadt, Germany). The experimental procedure is illustrated in Figure 2.

Experiments were conducted in amber-glass vials (internal dimensions: ⌀ 25 mm × 40 mm height). Each vial contained 75 mg of catalyst and 150 μL of a 1000 mg L^−1^ RB5 or BG stock solution, with the total volume adjusted to 15 mL using deionized water. This yielded a catalyst loading of 5 mg mL^−1^ and an initial dye concentration of 10 mg L^−1^. Mechanical agitation was provided by PTFE-coated or borosilicate-glass-coated magnetic stirring bars (⌀ 3 mm × 15 mm). Before each experiment, the suspensions were homogenized at 100 rpm for 1 min and maintained without stirring in darkness for 24 h to establish adsorption–desorption equilibrium.

The suspensions were stirred in darkness for 24 h using a programmable 15-position magnetic stirrer with three independently controlled groups (MS-T-S15, DLAB Scientific Co., Ltd., Beijing, China). Unless otherwise stated, experiments were conducted at 400 rpm, with 1 mL aliquots collected every 4 h. Each aliquot was centrifuged at 10,000 rpm for 5 min, after which 750 μL of the supernatant was transferred to a 10 mm-path-length microvolume cuvette. UV—Vis spectra were recorded over 320–1100 nm using an SP-V1100 spectrophotometer (DLAB Scientific Co., Ltd.). After analysis, the supernatant and sediment were recombined, resuspended, and returned to the reaction vial. All experiments were performed in triplicate. The pH of the initial dye solutions and the suspensions after tribocatalysis was measured using an MW150 MAX pH meter (Milwaukee Electronics Kft., Szeged, Hungary).

The spectra were baseline-corrected using the 1000–1100 nm region and restricted to 320–800 nm. Relative dye concentrations were determined from the integrated absorbance according to C_t_/C_0_ = A_t_/A_0_, where A_0_ and A_t_ denote the integrated absorbances after adsorption equilibration and after stirring for time t, respectively. Dye removal was calculated as

(1) $$\mathrm{Removal}\left(\%\right)=\left(1-\frac{C_{t}}{C_{0}}\right)\times 100.$$

Apparent pseudo-first-order rate constants (k_app_) were determined by linear regression of −ln(C_t_/C_0_) against stirring time.

The effects of stirring rate and RE-modification were investigated at 200, 400, and 800 rpm. Radical scavenger assay was conducted at 400 rpm in the presence of 5 mM Na_2_EDTA, DMSO, or 2-propanol (IPA). Catalyst recycling was evaluated at 800 rpm by centrifuging the suspension after each cycle and adding a fresh dye solution without washing or otherwise regenerating the recovered catalyst.

Chemical oxygen demand (COD) was measured before and after 24 h of treatment at 800 rpm using LCK 500 dichromate cuvette tests (0–150 mg L^−1^). Aliquots of 2.0 mL were digested at 148 °C for 2 h in an LT200 dry thermostat and subsequently analyzed using a DR1900 spectrophotometer with its pre-installed COD method. All COD reagents and equipment were supplied by Hach Company (Loveland, CO, USA), and the measurements were performed against a deionized water blank.

## 3. Results

### 3.1. Characterization of Pristine and Rare-Earth Modified ZnO Nanorods

**Figure 3:**
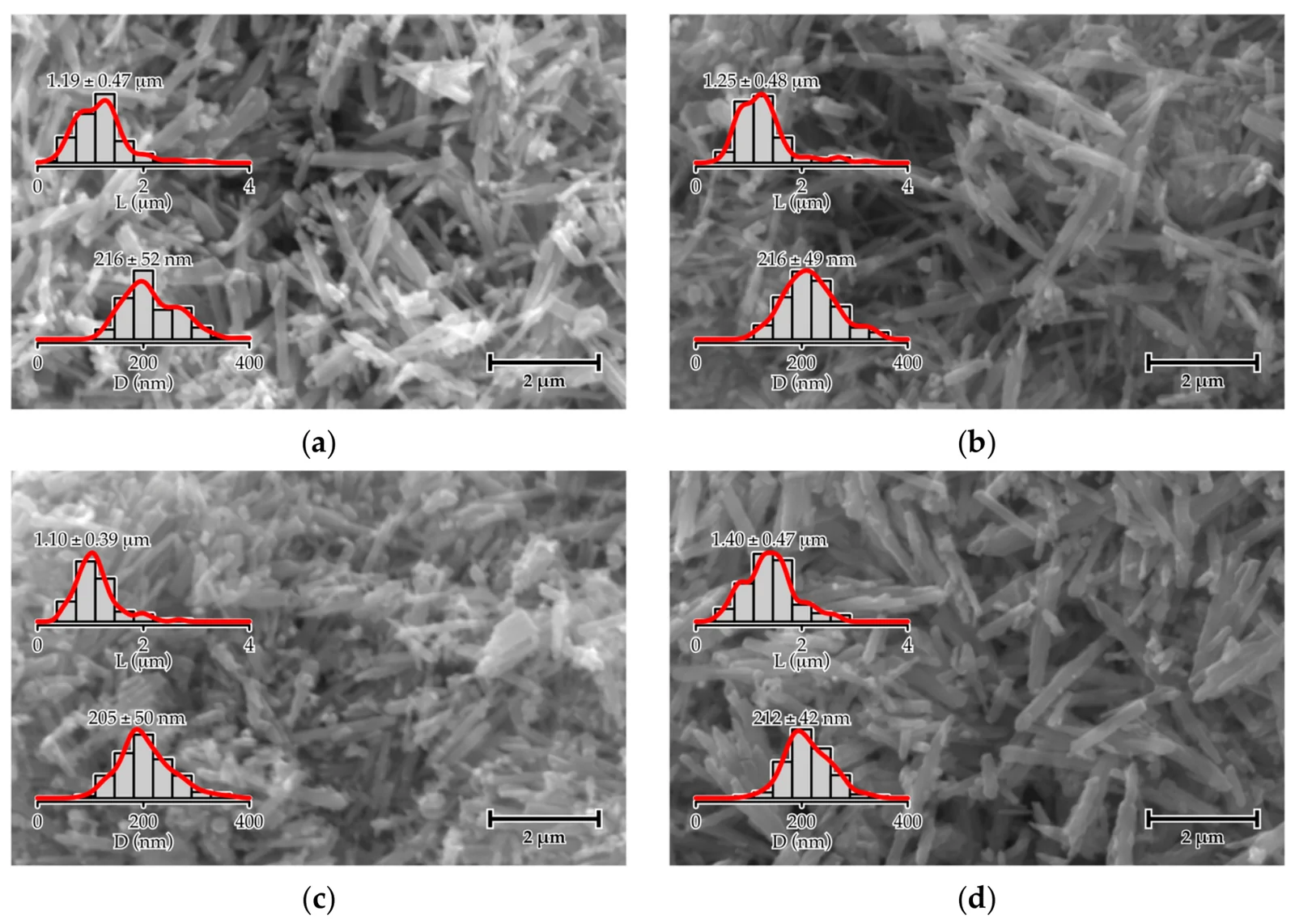
SEM micrographs and corresponding rod-length (*L*) and diameter (*D*) distributions for (**a**) Pristine ZnO. (**b**) Gd:ZnO. (**c**) Dy:ZnO. (**d**) Er:ZnO.

Figure 3a–d presents the morphology of pristine and rare-earth-modified ZnO obtained by the thermal decomposition of zinc acetate dihydrate, Zn(CH_3_COO)_2_.2H_2_O (ZAD), at 400 °C, followed by incipient-wetness modification with Gd, Dy, or Er. All samples exhibited predominantly rod-like structures, accompanied by aggregates and smaller rod fragments, commonly observed in ZnO powders prepared by the thermal decomposition of ZAD [35,36,37,38]. The rod-length (L) and diameter (D) distributions, obtained from 100 measurements, are inset in Figure 3a–d. The mean lengths were 1.19 ± 0.47, 1.25 ± 0.48, 1.10 ± 0.39, and 1.40 ± 0.47 µm for pristine ZnO, Gd:ZnO, Dy:ZnO, and Er:ZnO, respectively, while the mean diameters remain comparatively consistent at approximately 205–216 nm. Thus, the mean aspect ratio (L/D) was largely preserved, with values of approximately 5.5, 5.8, 5.4, and 6.6, respectively. Although marginally longer rods were measured for Er:ZnO, the difference was small relative to the broad size distributions and may reflect sampling variability. Overall, the results demonstrate that the incipient-wetness rare-earth (RE) modification did not degrade or substantially alter the original ZnO rod morphology.

**Figure 4:**
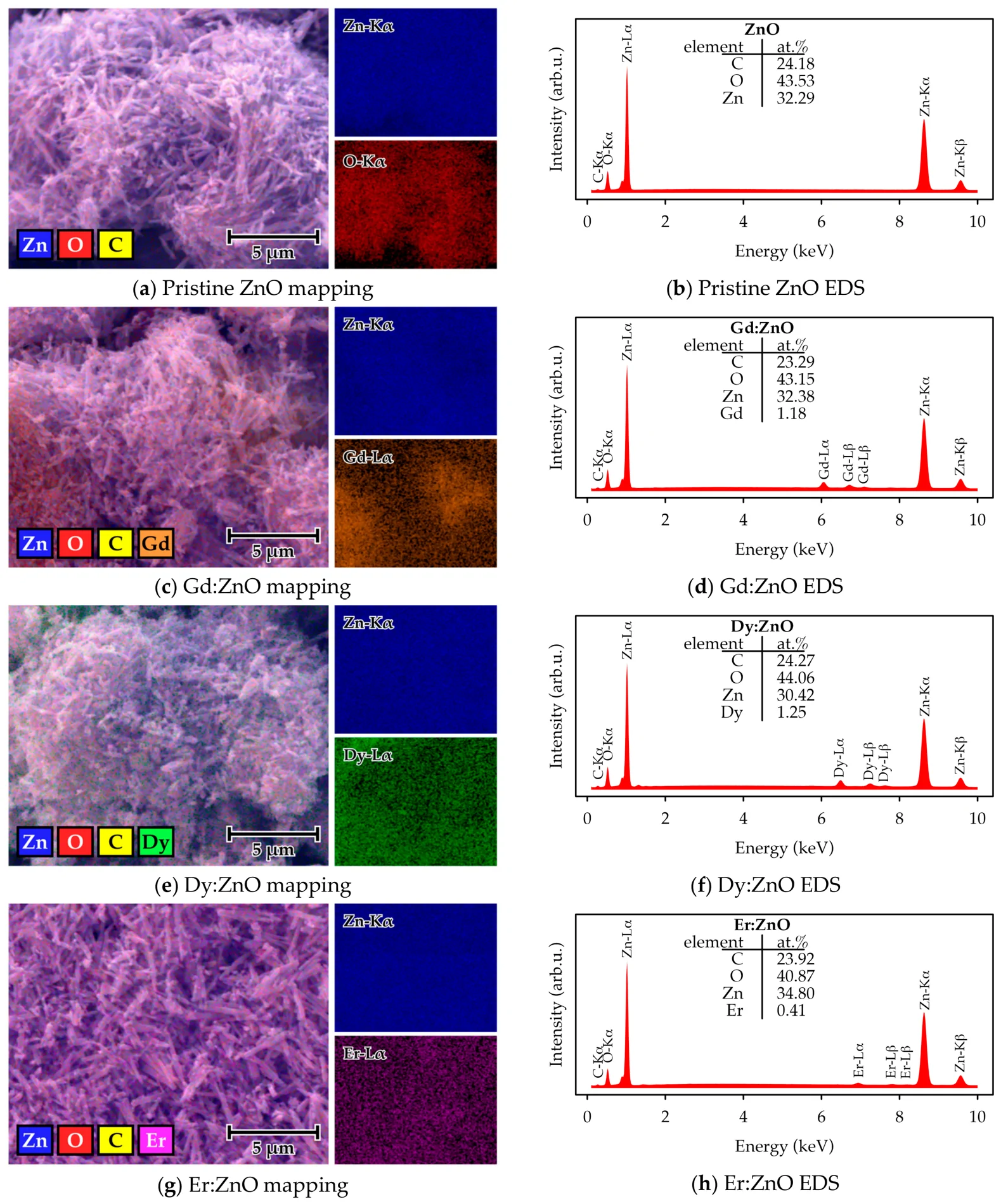
EDS mapping and the corresponding EDS spectra for (**a**,**b**) Pristine ZnO. (**c**,**d**) Gd:ZnO. (**e**,**f**) Dy:ZnO. (**g**,**h**) Er:ZnO.

The elemental composition of the samples was examined by SEM-EDS. The elemental maps and corresponding EDS spectra are presented in Figure 4 for pristine ZnO (Figure 4a,b), Gd:ZnO (Figure 4c,d), Dy:ZnO (Figure 4e,f), and Er:ZnO (Figure 4g,h). In all cases, the spectra indicated the high purity of the prepared catalysts, with Zn and O as the principal elements, together with the respective RE element in the modified samples and a C signal associated with sample preparation.

The presence of Gd, Dy, and Er was clearly distinguishable in the spectra of the respective RE-modified ZnO catalysts (Figure 4d,f,h), with measured contents of 1.18 at.% for Gd:ZnO, 1.25 at.% for Dy:ZnO, and 0.41 at.% for Er:ZnO. Normalization relative to the total cation content, expressed as RE/(RE + Zn) yielded 3.5 at.% Gd, 3.9 at.% Dy, and 1.2 at.% Er. The lower apparent Er content may reflect the limited sampled volume, particle geometry and the comparatively weak Er signal.

**Figure 5:**
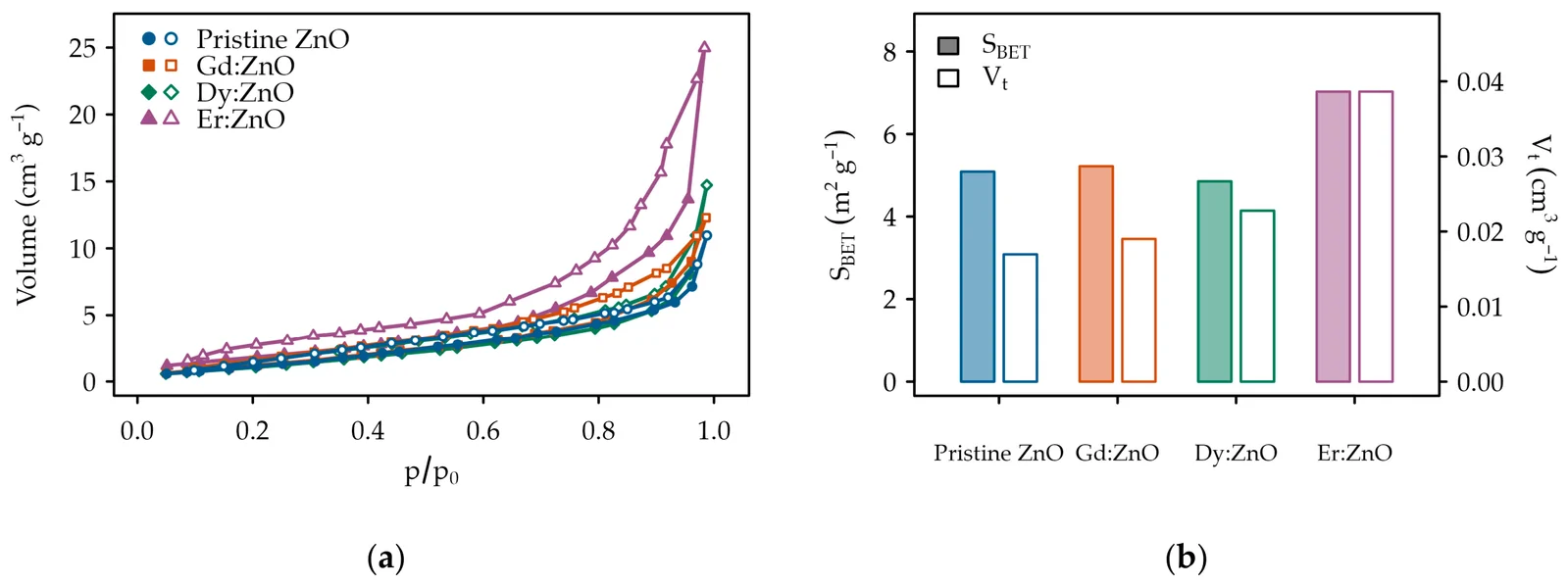
Nitrogen sorption results for the pristine and RE-modified ZnO samples: (**a**) Adsorption–desorption isotherms, where filled and open symbols represent the adsorption and desorption branches, respectively. (**b**) Calculated BET specific surface areas (*S_BET_*) and total pore volumes (*V_t_*).

To determine whether the preserved rod morphology was accompanied by comparable textural properties, the samples were further examined by nitrogen sorption. The nitrogen sorption isotherms of the samples are presented in Figure 5a. All samples exhibited type II isotherms with H3-like hysteresis and pronounced nitrogen uptake at high relative pressures. The specific surface area (*S_BET_*) was calculated by the Brunauer–Emmett–Teller (BET) method over *p*/*p*_0_ ≈ 0.05–0.30, while the total pore volume (*V_t_*) was estimated at the measured pressure closest to *p*/*p*_0_ = 0.99 (Figure 5b). Pristine ZnO, Gd:ZnO and Dy:ZnO had similarly low *S_BET_* values of 4.9–5.2 m^2^ g^−1^, whereas Er:ZnO exhibited a moderately higher value of 7.0 m^2^ g^−1^ and the highest *V_t_*, which increased from 0.017 cm^3^ g^−1^ for pristine ZnO to 0.039 cm^3^ g^−1^ for Er:ZnO.

BJH analysis did not reveal a clearly resolved pore-size distribution and is therefore not presented. The observed hysteresis is attributed primarily to interparticle voids between aggregated nanorods rather than to an internal mesoporous structure [39].

**Figure 6:**
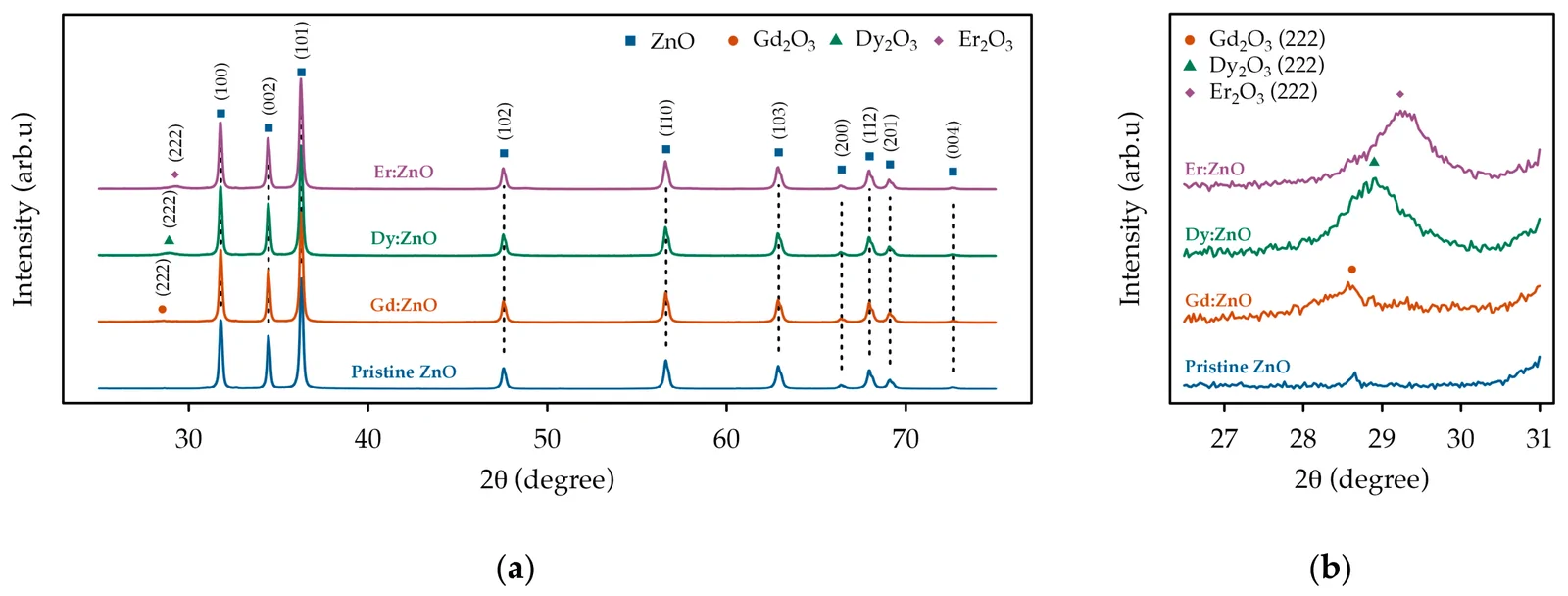
XRD characterization of the pristine and RE-modified ZnO nanorods: (**a**) Diffraction patterns over the 2θ range of 25–75°, showing the characteristic reflections of hexagonal wurtzite ZnO. (**b**) Enlarged 27–31° region showing the weak (222) reflections assigned to cubic Gd_2_O_3_, Dy_2_O_3_, and Er_2_O_3_ phases.

The phase composition and crystallinity of the samples were subsequently examined by XRD. XRD patterns of the as-prepared pristine and RE-modified ZnO nanorods are presented in Figure 6a.

Regardless of RE modification, the ZnO phase exhibited the hexagonal wurtzite structure, with characteristic reflections at 2θ = 31.8°, 34.5°, 36.3°, 47.6°, 56.6°, 62.9°, 66.4°, 68.0°, 69.1°, and 72.6°, assigned to the (100), (002), (101), (102), (110), (103), (200), (112), (201), and (004) planes, respectively (JCPDS card no. 36-1451) [40,41]. The unit-cell parameters obtained by Rietveld refinement, together with the mean crystallite size and microstrain, obtained by size-strain analysis, are summarized in Table 1.

Size–strain analysis of the ZnO (100), (002), (101), (102), (110), and (103) reflections yielded mean crystallite sizes of 62.8, 67.1, 65.7, and 64.7 nm for pristine ZnO, Gd:ZnO, Dy:ZnO, and Er:ZnO, respectively. These values are consistent with the formation of polycrystalline ZnO nanorods, obtained at 400 °C [42], and indicate that the subsequent RE modification caused no appreciable recrystallization of the ZnO phase. The ZnO unit-cell parameters showed only minor variations, while the microstrain remained similar across all samples (0.234–0.236%).

Despite the low RE content, weak reflections attributable to the corresponding RE oxide phases were detected between 2θ = 27–31°, as shown in the enlarged region in Figure 6b. The reflections centered at 28.6°, 28.9°, and 29.2° for Gd:ZnO, Dy:ZnO, and Er:ZnO, respectively, were consistent with the (222) reflections of cubic Gd_2_O_3_ (JCPDS card no. 86-2477) [43], Dy_2_O_3_ (JCPDS card no. 86-1327) [44], and Er_2_O_3_ (JCPDS card no. 77-0777) [45]. Rietveld refinement yielded corresponding RE_2_O_3_ phase contents of 2.18, 2.48, and 2.45 vol.%, respectively (Table 1). The crystalline RE_2_O_3_ phase fractions correspond to approximate RE/(RE + Zn) values of 1.3, 1.5, and 1.6 at.% for Gd:ZnO, Dy:ZnO, and Er:ZnO, respectively, with precision limited by the weak, broad RE_2_O_3_ reflections. Scherrer analysis of the (222) reflections gave approximate RE-oxide crystallite sizes of 10–11 nm, indicating the presence of nanocrystalline RE-oxide phases in the modified samples. The results from the XRD characterization support the formation of mixed-phase RE_2_O_3_/ZnO composites, although limited incorporation of RE ions into the ZnO lattice cannot be excluded.

**Figure 7:**
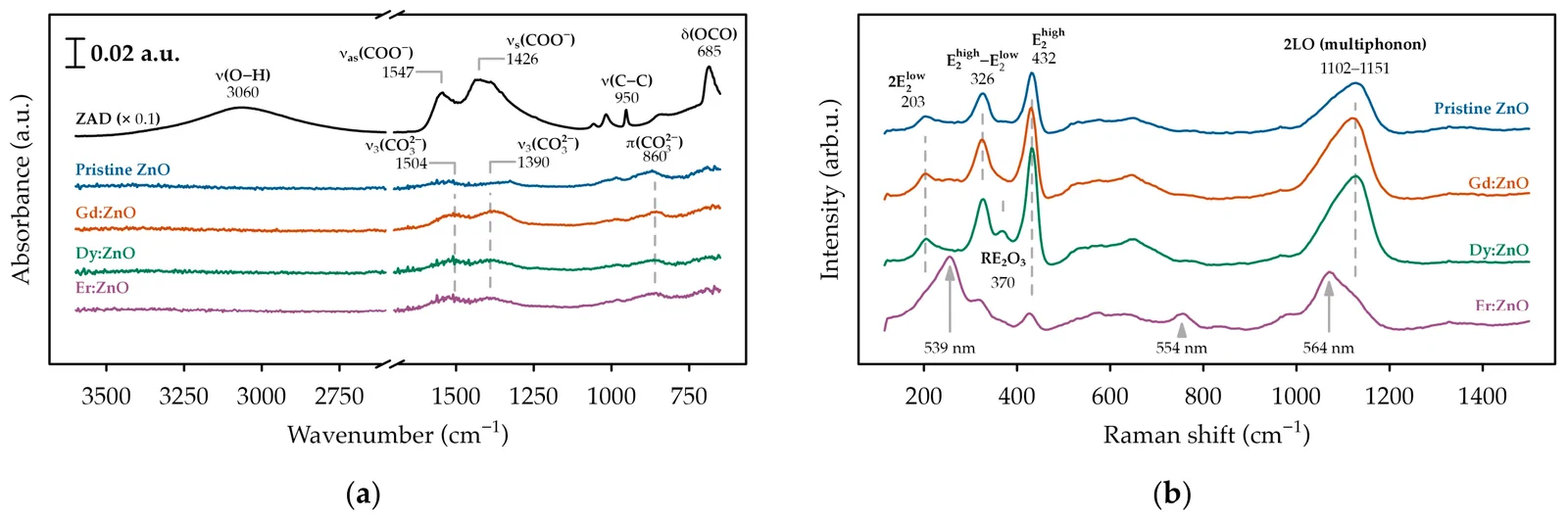
Vibrational spectroscopy of pristine and RE-modified ZnO nanorods: (**a**) ATR-FTIR spectra of the ZAD and the as-prepared tribocatalysts. (**b**) Raman spectra of pristine and RE-modified ZnO nanorods. Arrows mark the Er^3+^ fluorescence features, expressed as emission wavelengths under 532 nm excitation.

The pristine and RE-modified ZnO powders were additionally characterized by vibrational spectroscopy (Figure 7). To confirm the decomposition of the zinc acetate precursor (ZAD) during thermal treatment, ATR-FTIR spectra of the ZAD and the pristine and RE-modified ZnO after calcination are shown in Figure 7a. The ZAD precursor is characterized by a broad ν(O-H) band centered at ≈3060 cm^−1^, together with characteristic acetate bands at 1547 and 1426 cm^−1^, assigned to ν_as_(COO^−^) and ν_s_(COO^−^), respectively [46]. The bands at approximately 950 and 685 cm^−1^ were attributed to ν (C–C) and δ(OCO), respectively [47].

The characteristic ZAD vibrational bands were completely suppressed in the ATR-FTIR spectra of the calcined powders, supporting the complete decomposition of the ZAD. However, it must be noted, that the RE-modified ZnO nanorods, in particular, exhibited weak bands at approximately 1504 and 1390 cm^−1^, which could be assigned to the split ν_3_(CO_3_^2−^) stretching mode, and in combination with the weak π(CO_3_^2−^) feature at approximately 860 cm^−1^, consistent with carbonate species [48].

Further information about the phase composition of the pristine and RE-modified ZnO powders was obtained by Raman spectroscopy (Figure 7b). Hexagonal wurtzite ZnO was confirmed by the characteristic Raman modes at 203 cm^−1^ ($2E_{2}^{\mathrm{low}}$), 326 cm^−1^ ($E_{2}^{\mathrm{high}}-E_{2}^{\mathrm{low}}$), and 432 cm^−1^ ($E_{2}^{\mathrm{high}}$) [30,49]. The broad feature extending from 1102 to 1151 cm^−1^ was assigned to second-order LO multiphonon scattering [50,51]. The characteristic ZnO features were preserved in the Gd:ZnO and Dy:ZnO spectra but were obscured in the Er:ZnO case by fluorescence features at apparent Raman shifts of 256 cm^−1^, 754, and 1070 cm^−1^. Under 532 nm excitation, these positions correspond to emission wavelengths of approx. 539, 554, and 564 nm, respectively, as indicated in Figure 7. They agree with the characteristic green Er^3+^ emission lines reported for Er_2_O_3_ and attributed to the ^4^S_3/2_ →^4^I_15/2_ transition [52]. The most intense Raman bands of the RE oxide phases are generally expected in the 360–380 cm^−1^ region [52,53] but were not resolved for Gd:ZnO or Er:ZnO, likely because of their low contents and weak Raman intensities, as well as strong fluorescence in the latter case. By contrast, the additional band at 370 cm^−1^ in the Dy:ZnO spectrum coincides with the F_g_ + A_g_ mode, reported for cubic Dy_2_O_3_ under 532 nm excitation [53].

**Figure 8:**
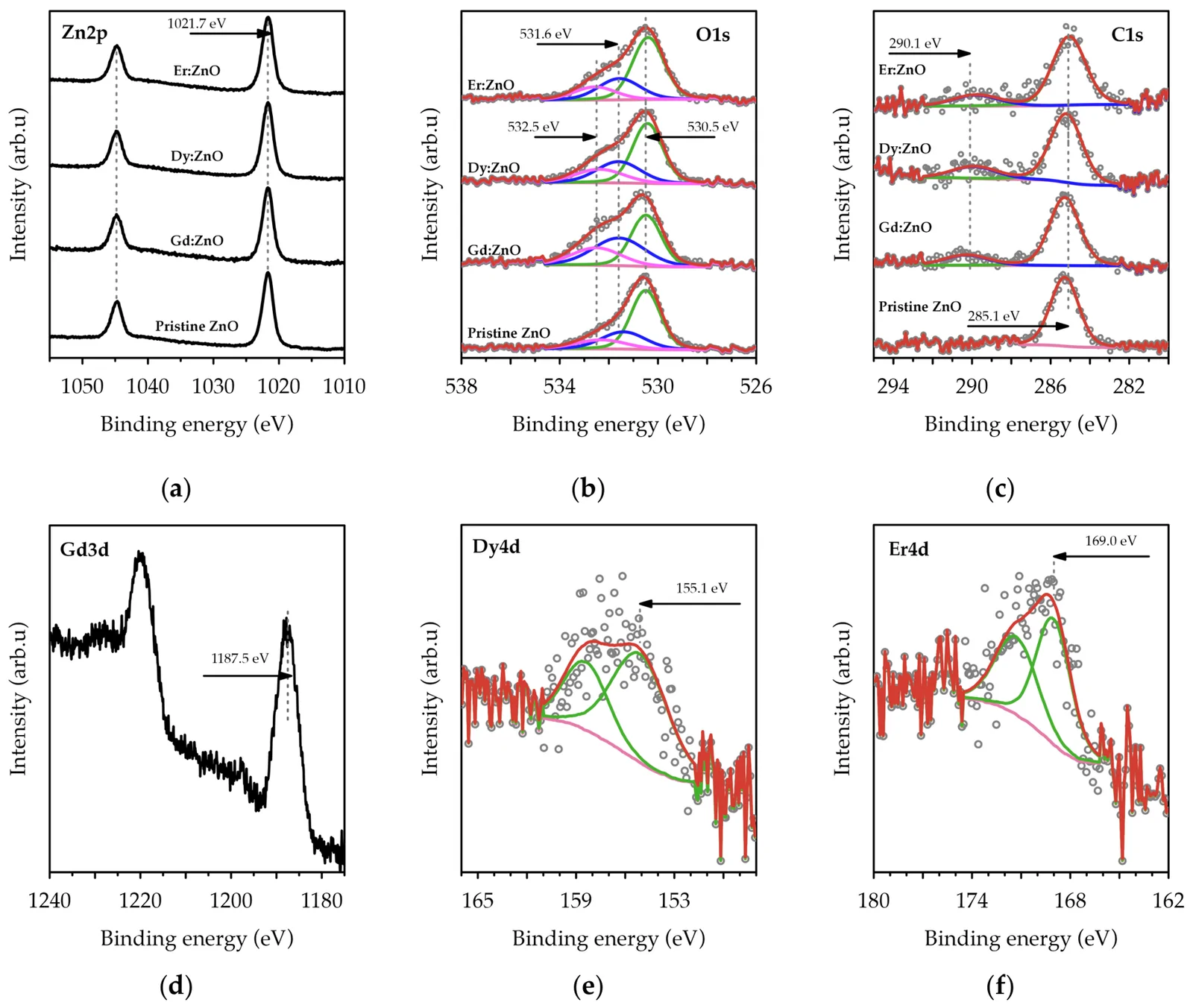
High-resolution XPS spectra of the pristine and RE-modified ZnO catalysts: (**a**) Zn 2p region for all catalysts. (**b**) O 1s region for all catalysts. (**c**) C 1s region for all catalysts. (**d**) Gd 3d region of Gd:ZnO. (**e**) Dy 4d region of Dy:ZnO. (**f**) Er 4d region of Er:ZnO.

The surface composition and chemical states of the pristine ZnO and RE-modified ZnO nanorods was examined by XPS. Figure 8 presents the high-resolution spectra of the Zn 2p, O 1s and C 1s regions for all samples (Figure 8a–c), together with the Gd 3d, Dy 4d and Er 4d regions of Gd:ZnO, Dy:ZnO and Er:ZnO, respectively (Figure 8d–f).

In the Zn 2p region (Figure 8a), peaks centered at approximately 1021.7 and 1044.8 eV were assigned to the Zn 2p_3/2_ and Zn 2p_1/2_ components, respectively, characteristic of Zn^2+^ [54,55].

The O 1s spectra (Figure 8b) comprised a broad envelope that was fitted with three contributions. The dominant component at approximately 530.5 eV was assigned to lattice O^2−^ [55,56]. The higher-binding-energy components at approximately 531.6 and 532.5 eV were associated with non-lattice oxygen and surface –OH species, as well as more weakly bound surface species, including adsorbed H_2_O and potentially oxygen-containing adventitious species, respectively [57,58]. The C 1s spectra (Figure 8c) were dominated by a peak at approximately 285.1 eV, assigned to C–C/C–H species arising predominantly from adventitious carbon.

The Zn 2p and O 1s regions did not undergo any substantial changes following RE modification. Nevertheless, the relative contribution of the non-lattice oxygen component increased slightly following RE modification and was highest for Gd:ZnO, indicating a change in the surface oxygen speciation. In the C 1s region, an additional contribution at ≈290.1 eV appeared in the RE-modified samples and was attributed to carbonate species [48], supporting the assignment of the carbonate bands observed in their ATR-FTIR spectra (Figure 7a).

The Gd 3d spectrum of Gd:ZnO is presented in Figure 8d. Peaks centered at approximately 1187.5 and 1220.0 eV were assigned to the Gd 3d_5/2_ and Gd 3d_3/2_ components, respectively. Their positions and spin–orbit separation of approximately 32.5 eV are consistent with Gd^3+^ [50,59]. The Dy 4d region of Dy:ZnO (Figure 8e) exhibited a broad, low-intensity signal fitted with contributions at approximately 155.1 and 158.4 eV. The position and spectral profile of this region are consistent with Dy being present predominantly in the Dy^3+^ state [60,61]. Finally, the Er 4d spectrum of Er:ZnO (Figure 8f) exhibited a principal contribution at approximately 169 eV, together with the associated higher-binding-energy spectral structure, consistent with Er^3+^ [30,62].

The surface compositions determined by XPS are summarized in Table 2. Recalculation of the RE content relative to the total metal content, using RE/(RE + Zn), yields approximately 6.8 at.% Gd for Gd:ZnO, 2.5 at.% Dy for Dy:ZnO and 5.8 at.% Er for Er:ZnO. The higher Gd and Er contents measured by XPS compared with EDS are consistent with surface enrichment of these modifiers, given the greater surface sensitivity of XPS.

In summary, thermal decomposition of ZAD produced polydisperse, predominantly rod-like ZnO particles with low specific surface areas, consistent with literature reports for this synthesis route. Subsequent incipient-wetness modification preserved the rod morphology, hexagonal wurtzite ZnO structure and ZnO crystallite size, while introducing nanocrystalline cubic Gd_2_O_3_, Dy_2_O_3_ and Er_2_O_3_ phases. ATR-FTIR and XPS additionally indicated the presence of surface carbonate species in the RE-modified samples. These results demonstrate successful RE-oxide modification of the ZnO nanorods without substantial alteration of the morphology, texture or underlying wurtzite structure of the parent catalyst.

### 3.2. Tribocatalytic Activity of the Pristine and Rare-Earth-Modified ZnO Nanorods

#### 3.2.1. Effects of Dye Charge and RE Modification

**Figure 9:**
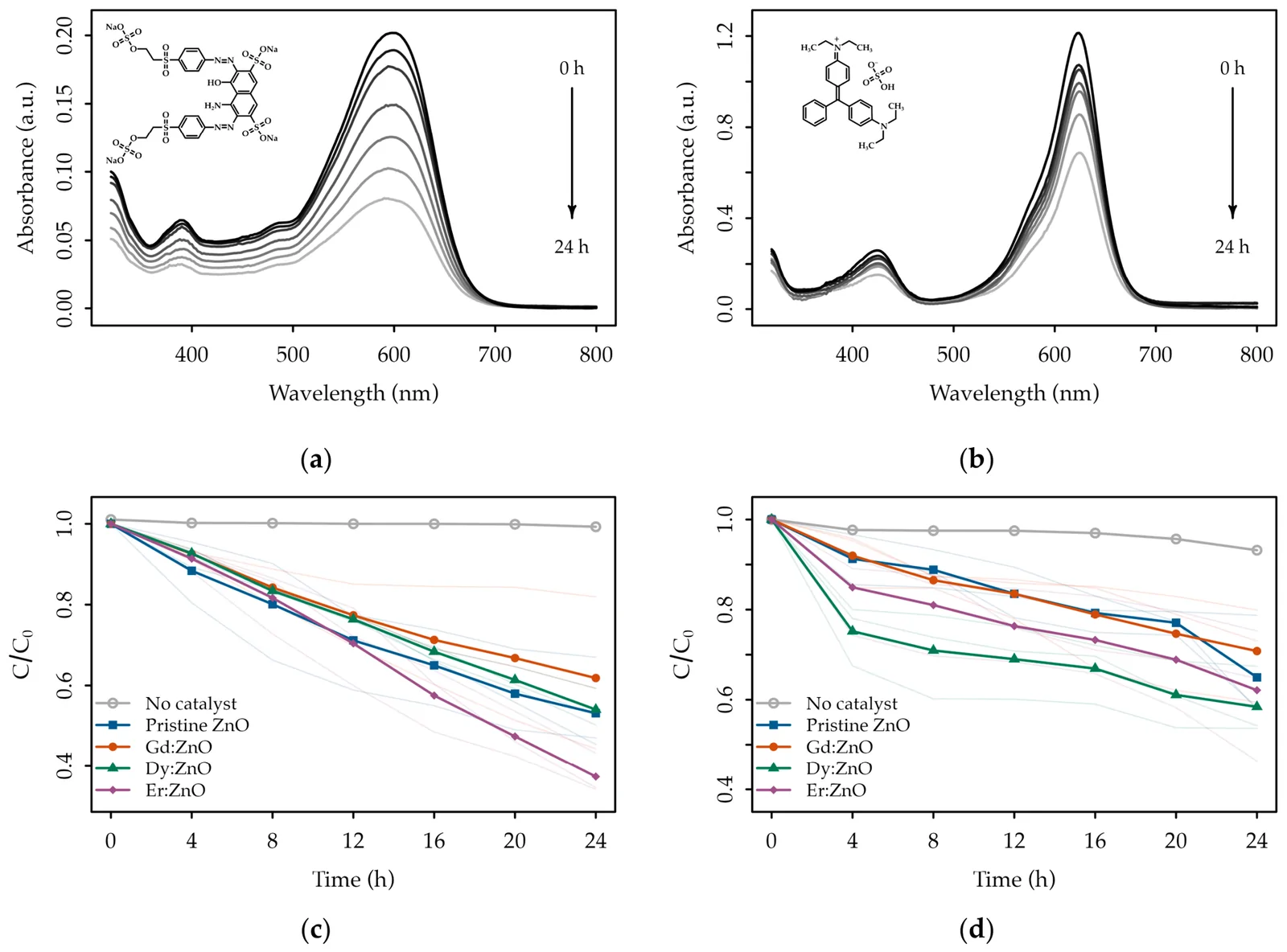
Tribocatalytic removal of RB5 and BG using pristine and RE-modified ZnO nanorods at 400 rpm: (**a**) RB5 spectral evolution with pristine ZnO. (**b**) BG spectral evolution with pristine ZnO. (**c**) RB5 concentration profiles. (**d**) BG concentration profiles. Insets in (**a**,**b**) show molecular structure of the dyes. Dense lines in (**c**,**d**) show means from three independent experiments, while faint lines show the individual runs.

The tribocatalytic activity of the pristine and RE-modified ZnO powders was evaluated in darkness using two model contaminants with different ionic characters: the anionic azo dye Reactive Black 5 (RB5) and the cationic triarylmethane dye Brilliant Green (BG). Figure 9a,b shows the spectral evolution of 10 mg L^−1^ RB5 and BG, respectively, during 24 h of treatment with pristine ZnO nanorods at a catalyst concentration of 5 mg mL^−1^. The decolorization of both dyes is evidenced by the continuous decrease in the intensity of their principal absorption bands, located at approximately 600 nm for RB5 and 625 nm for BG. The corresponding concentration profiles obtained using pristine and RE-modified ZnO nanorods are presented in Figure 9c,d.

The removal efficiency depended on both the dye and the catalyst composition. For RB5, a progressive decrease in concentration was observed with all four materials. After 24 h at 400 rpm, pristine ZnO achieved a removal efficiency of 47 ± 7%. Comparable values of 38 ± 11% and 46 ± 3% were obtained with Gd:ZnO and Dy:ZnO, respectively, whereas Er:ZnO exhibited the highest RB5 removal efficiency of 63 ± 3%. This represents an increase of 16 percentage points, or approximately 34%, relative to pristine ZnO. The BET surface area of Er was approximately 37% higher than that of pristine ZnO (7.0 vs 5.1 m^2^ g^−1^), which may partly account for the similar relative increase in RB5 removal. However, no corresponding relationship between surface area and activity was observed for Gd and Dy. The similar relative increases in BET surface area and RB5 removal indicate that the larger surface area of Er:ZnO could account for much of its enhanced performance. Although nanoscale Er_2_O_3_ has itself been reported to exhibit tribocatalytic activity [63], its contribution cannot be separated from that of the increased surface area in the present experiments.

Lower removal efficiencies were generally obtained for BG. Pristine ZnO removed 35 ± 7% of the dye, while efficiencies of 29 ± 6%, 42 ± 5%, and 38 ± 8% were obtained with Gd:ZnO, Dy:ZnO, and Er:ZnO, respectively. RE modification therefore had a comparatively modest effect on BG removal, with Dy:ZnO exhibiting the highest mean efficiency. In the catalyst-free controls, stirring with the PTFE-sealed bar resulted in only 1% removal of RB5 and 7% removal of BG. RB5 removal exceeded BG removal by 4–25 percentage points across the four catalysts, indicating more favorable interaction of the anionic dye with the ZnO-based surfaces under the employed conditions.

The initial RB5 and BG solutions had pH values of 5.78 ± 0.08 and 5.89 ± 0.20, respectively. After tribocatalysis, the pH values of the RB5 suspensions were 6.01 ± 0.31, 6.42 ± 0.03, 6.56 ± 0.09, and 6.77 ± 0.10 for pristine ZnO, Gd:ZnO, Dy:ZnO, and Er:ZnO, respectively. The corresponding values for BG were 7.39 ± 0.33, 7.09 ± 0.20, 7.11 ± 0.22, and 7.11 ± 0.19. These pH values are below the experimental isoelectric point of 9.4 reported for ZnO nanorods by Baillard et al. [64], suggesting that positively charged ZnO surfaces could favour electrostatic attraction of anionic RB5 over cationic BG.

This ordering differs from that reported by Chong et al., who obtained higher tribocatalytic removal of cationic methylene blue than of anionic methyl orange using ZnO [17]. Che et al. similarly obtained higher removal of cationic crystal violet than of the anionic dyes Acid Orange 7 and methyl orange using Er_2_O_3_, although the comparatively low removal of cationic Methylene blue in the same study showed that ionic character alone did not determine the activity order [63]. Dye-dependent activity has also been reported for ZnO photocatalysis, e.g., Motelica et al. observed lower removal of cationic Gentian violet than of cationic Methylene blue and anionic Methyl orange using a ZnO photocatalyst [65]. The present difference between RB5 and BG can therefore be associated with the combined effects of dye charge, molecular structure, and affinity for the catalyst surface.

#### 3.2.2. Effects of the Friction Pair and Reactive-Species Scavengers

**Figure 10:**
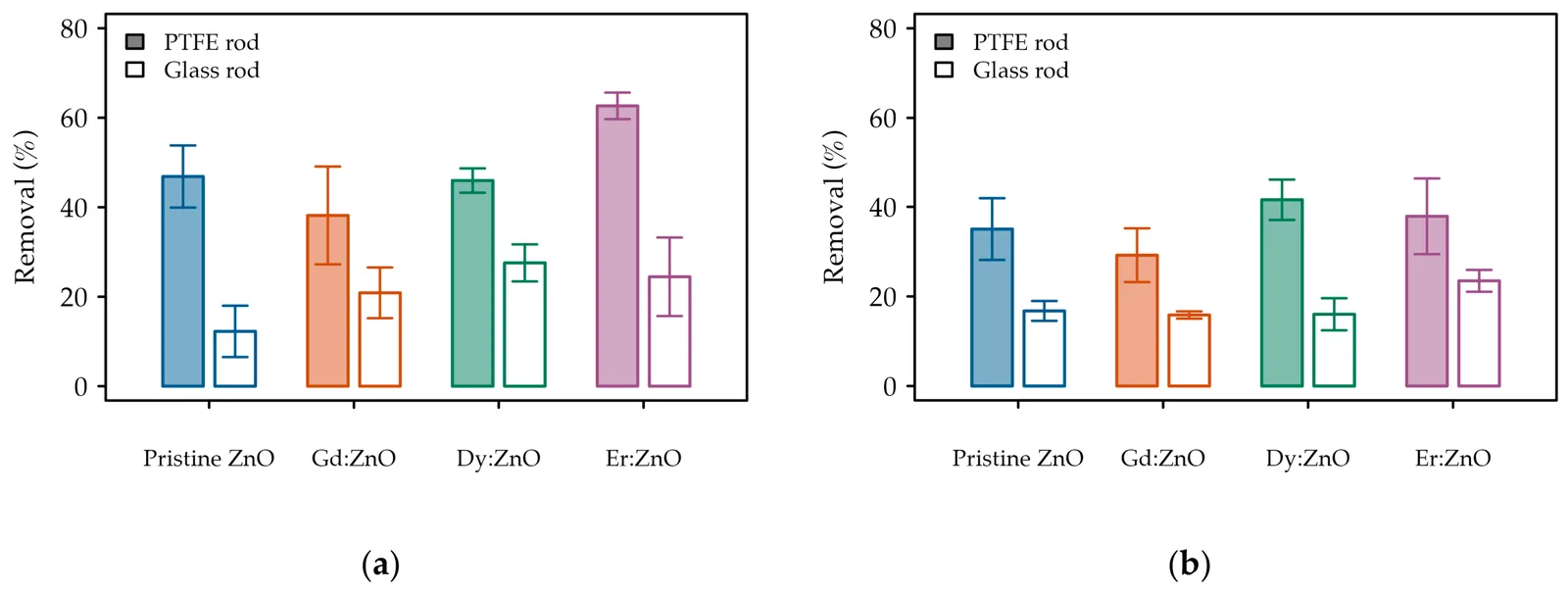
Comparison of the removal efficiencies obtained using PTFE- and glass-sealed stirring bars at 400 rpm: (**a**) Removal of RB5 using pristine and RE-modified ZnO nanorods. (**b**) Removal of BG using pristine and RE-modified ZnO nanorods. Bars represent mean values from three independent experiments; error bars indicate standard deviations.

The effect of the friction pair was investigated by comparing the removal efficiencies obtained using PTFE- and glass-sealed stirring bars. As shown in Figure 10, the glass-sealed bar produced lower removal efficiencies for all four catalysts and both dyes. For RB5 (Figure 10a), the removal efficiencies decreased to 12 ± 6%, 21 ± 6%, 28 ± 4%, and 24 ± 9% for pristine ZnO, Gd:ZnO, Dy:ZnO, and Er:ZnO, respectively.

On average, these values were approximately 57% lower than those obtained using the PTFE-sealed bar. Similarly, the removal efficiencies of BG decreased to 17 ± 2%, 16 ± 1%, 16 ± 4%, and 23 ± 2%, respectively (Figure 10b), corresponding to an average decrease of approximately 50%.

Li et al. similarly observed substantially lower dye removal with glass-sealed than with PTFE-sealed stirring rods and proposed that coating hardness influenced mechanical-energy transfer during friction [15]. Together with previous findings on ZnO nanorods and other ZnO morphologies [16,17], the present results support an important role of frictional contact. However, the effects of contact conditions and mechanical-energy transfer cannot be separated from those of friction-induced charge generation in this comparison.

**Figure 11:**
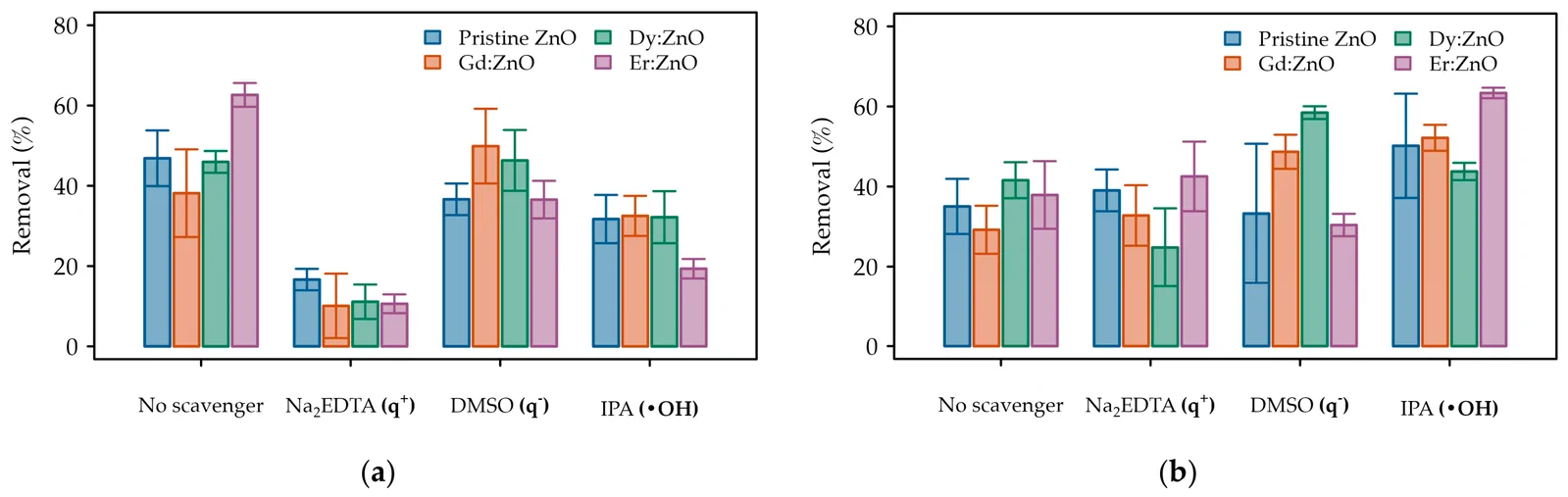
Effect of reactive-species scavengers on dye removal by pristine and RE-modified ZnO nanorods: (**a**) Removal of RB5 in the absence and presence of scavengers. (**b**) Removal of BG in the absence and presence of scavengers. Bars represent mean values from three independent experiments; error bars indicate standard deviations.

To probe the involvement of charged and reactive species in dye removal, Na_2_EDTA, DMSO, and IPA were added as scavengers assigned to positive charges, negative charges, and •OH, respectively. These compounds are conventionally used to probe photogenerated h^+^, e^−^, and •OH in photocatalysis [66], and a similar approach has been adopted in tribocatalytic studies by assigning the scavenged species to friction-generated q^+^ and q^−^ [63]. The latter notation is therefore used here. Because scavengers may also interact with other reactive species, the dye, or the catalyst surface, their effects provide qualitative indications of the predominant reaction pathways rather than exclusive identification of individual species [66,67]. For RB5 (Figure 11a), Na_2_EDTA markedly decreased the removal efficiency of all four materials. IPA also inhibited RB5 removal in every case, with the largest decrease observed for Er:ZnO. By contrast, DMSO produced a less systematic response: RB5 removal decreased with pristine ZnO and Er:ZnO, remained nearly unchanged with Dy:ZnO, and increased slightly with Gd:ZnO. The consistent inhibition produced by Na_2_EDTA and IPA supports a predominantly oxidative RB5-removal pathway involving q^+^ and •OH-derived species, in agreement with scavenger experiments reported for tribocatalytic Er_2_O_3_ [63].

A markedly different response was observed for BG (Figure 11b). Na_2_EDTA produced only limited changes for pristine ZnO, Gd:ZnO, and Er:ZnO but decreased the removal efficiency of Dy:ZnO. DMSO increased BG removal with Gd:ZnO and Dy:ZnO, had little effect on pristine ZnO, and decreased the activity of Er:ZnO. IPA increased the apparent BG removal efficiency for all four materials, with the largest increase observed for Er:ZnO. Such enhancement in the presence of an alcohol is not unprecedented, because alcohols may interact with positive charges and catalyst surfaces or generate secondary organic radicals rather than acting exclusively as •OH scavengers [66,67]. Together with the weaker interaction of BG with the catalyst surface, the absence of a common inhibitory response is consistent with a greater contribution from solution-phase reactive intermediates to BG removal.

Taken together, the results show that the activity of pristine and RE-modified ZnO depends on the catalyst composition, friction-pair material, and identity of the dye. RB5 exhibited greater overall removal and a systematic response to q^+^ and •OH scavengers, supporting a predominantly oxidative pathway involving surface-associated charged and reactive species. BG showed lower removal efficiencies and strongly material-dependent scavenger effects, consistent with a larger contribution from solution-phase intermediates. Among the RE-modified materials, Er:ZnO exhibited the highest activity toward RB5, while Dy:ZnO provided the highest mean removal of BG under the standard conditions.

#### 3.2.3. Effect of Stirring Rate on Dye-Removal Kinetics

**Figure 12:**
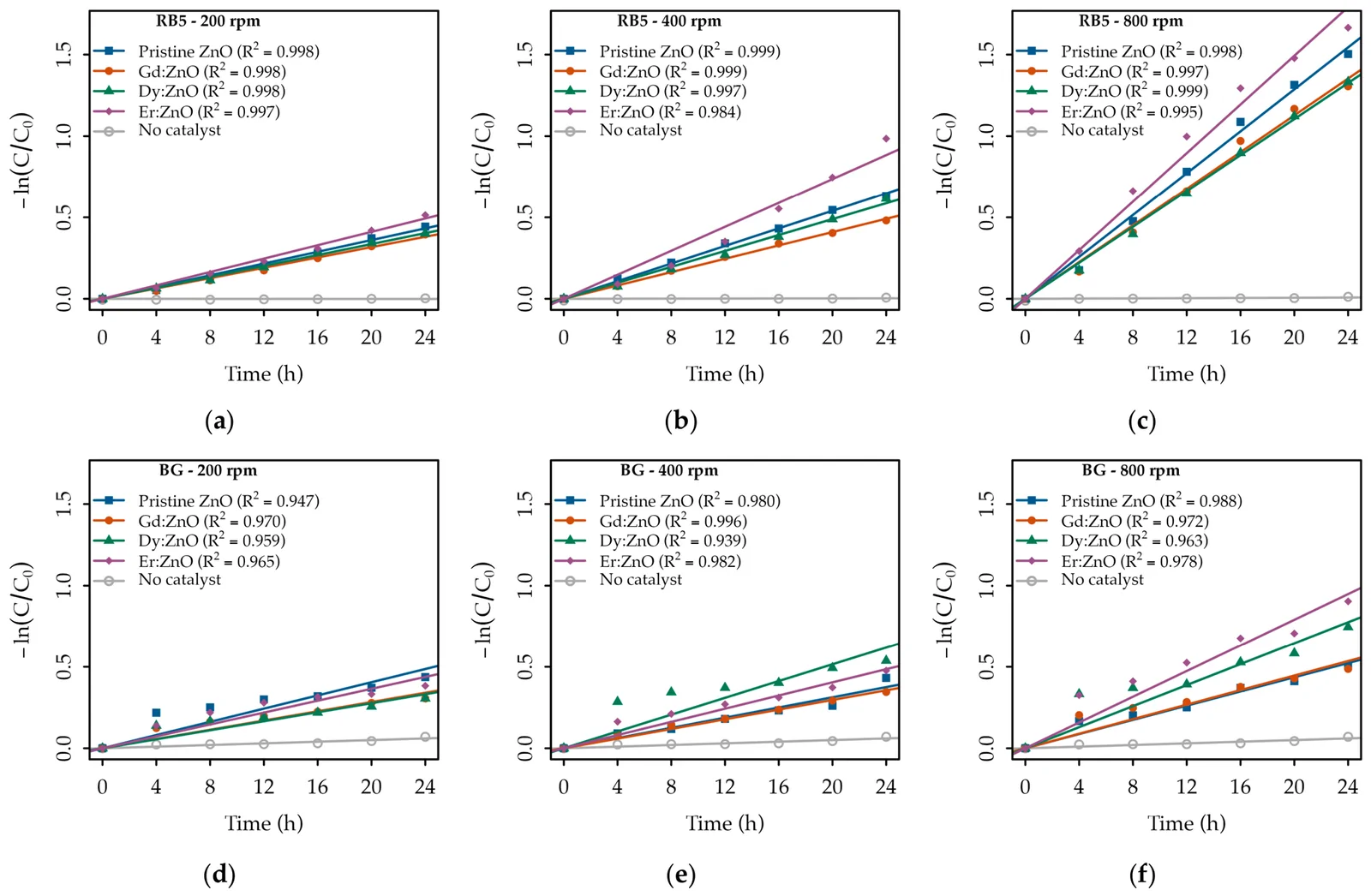
Pseudo-first-order kinetic plots for dye removal using pristine and RE-modified ZnO nanorods with a PTFE-sealed stirring bar: (**a**) RB5 removal at 200 rpm. (**b**) RB5 removal at 400 rpm. (**c**) RB5 removal at 800 rpm. (**d**) BG removal at 200 rpm. (**e**) BG removal at 400 rpm. (**f**) BG removal at 800 rpm. The results represent mean values from three independent experiments; error bars are omitted for clarity. Lines show linear fits to the mean data, with corresponding R^2^ values given in the legends.

To examine the influence of stirring rate and the resulting increase in mechanical interaction on dye-removal kinetics, experiments were conducted at 200, 400, and 800 rpm using pristine and RE-modified ZnO nanorods. The concentration profiles were linearized according to the pseudo-first-order kinetic model, −ln(C/C_0_) = k_app_t, where k_app_ is the apparent rate constant. The resulting kinetic plots are presented in Figure 12a–c for RB5 and Figure 12d–f for BG.

For RB5, increasing the stirring rate produced a pronounced and consistent increase in the slopes of the linearized concentration profiles (Figure 12a–c). The differences among the materials were relatively small at 200 rpm but became more apparent at 400 and 800 rpm. Er:ZnO exhibited the steepest slope at each stirring rate, in agreement with its higher RB5 removal efficiency. At 800 rpm, the removal kinetics were substantially enhanced for all four catalysts, while the catalyst-free control remained nearly unchanged. Similar increases in tribocatalytic activity with stirring rate have been reported for ZnO nanorods and Er_2_O_3_ nanoparticles and have been attributed to the increased frequency of frictional contact between the catalyst, stirring element, and reactor surface [16,63].

The BG kinetic profiles exhibited lower slopes and greater deviations from linearity than those obtained for RB5 (Figure 12d–f). The influence of stirring rate was also more dependent on catalyst composition. The increases observed for pristine ZnO and Gd:ZnO were comparatively modest, whereas Dy:ZnO and Er:ZnO showed clearer enhancement at higher stirring rates. At 800 rpm, Er:ZnO exhibited the highest apparent rate constant and BG removal efficiency, followed by Dy:ZnO. The weaker linearity and greater material dependence observed for BG are consistent with the more complex response indicated by the scavenger experiments.

**Figure 13:**
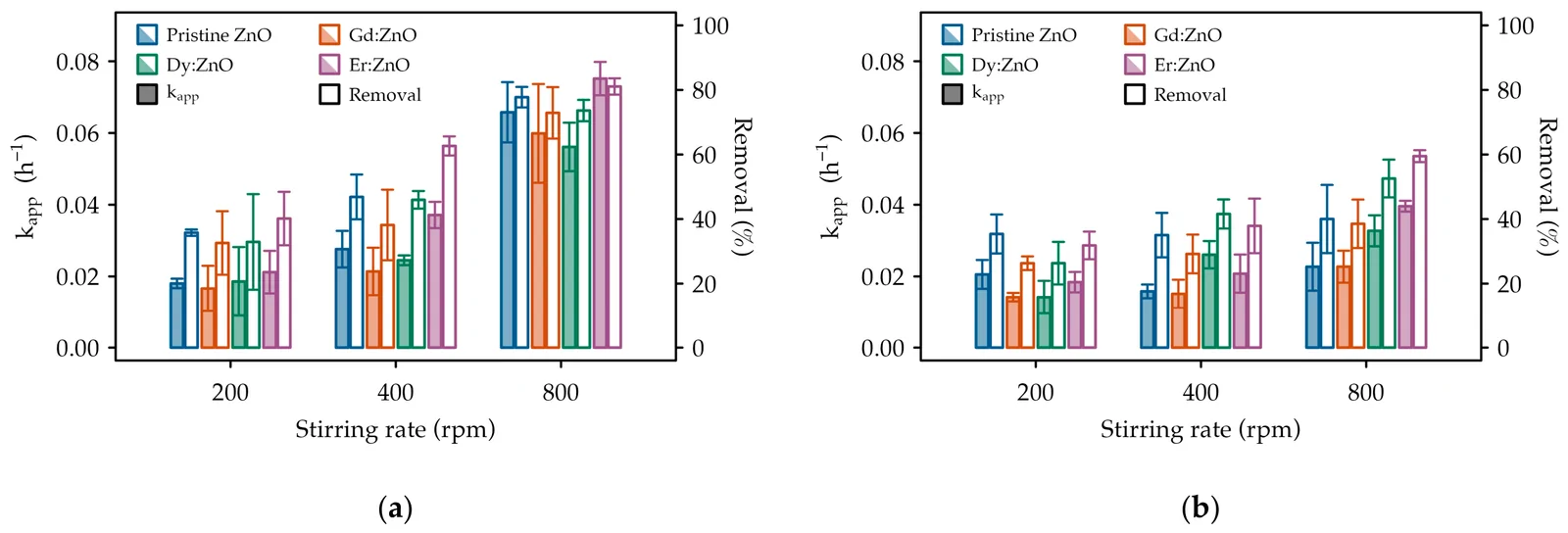
Comparison of the apparent rate constants (k_app_) and dye-removal efficiencies obtained using pristine and RE-modified ZnO nanorods at 200, 400, and 800 rpm: (**a**) RB5. (**b**) BG. Filled and open bars represent k_app_ and removal efficiency, respectively. Bars show mean values from three independent experiments, with error bars indicating standard deviations; k_app_ was determined separately for each run.

Apparent pseudo-first-order rate constants (k_app_) were determined by linear regression of −ln(C/C_0_) against stirring time for each independent run. Their mean values and standard deviations are compared with the corresponding removal efficiencies after 24 h in Figure 13. As expected for measurements performed over the same reaction period, increases in k_app_ were generally accompanied by increases in the final removal efficiency.

For RB5 (Figure 13a), both k_app_ and the removal efficiency increased markedly with stirring rate for all four catalysts. Er:ZnO was the most active material throughout the investigated range, although the differences among the catalysts became comparatively small at 800 rpm. In contrast, the effect of stirring rate on BG removal was less uniform (Figure 13b). Dy:ZnO and Er:ZnO showed the clearest increases in k_app_ and removal efficiency, particularly at 800 rpm, whereas the changes observed for pristine ZnO and Gd:ZnO were less pronounced. Thus, increased mechanical interaction enhanced the removal of both dyes, but the magnitude of the response depended on the catalyst composition and was considerably more uniform for RB5 than for BG.

#### 3.2.4. Catalyst Reusability and Dye Mineralization Assessed by COD Removal

**Figure 14:**
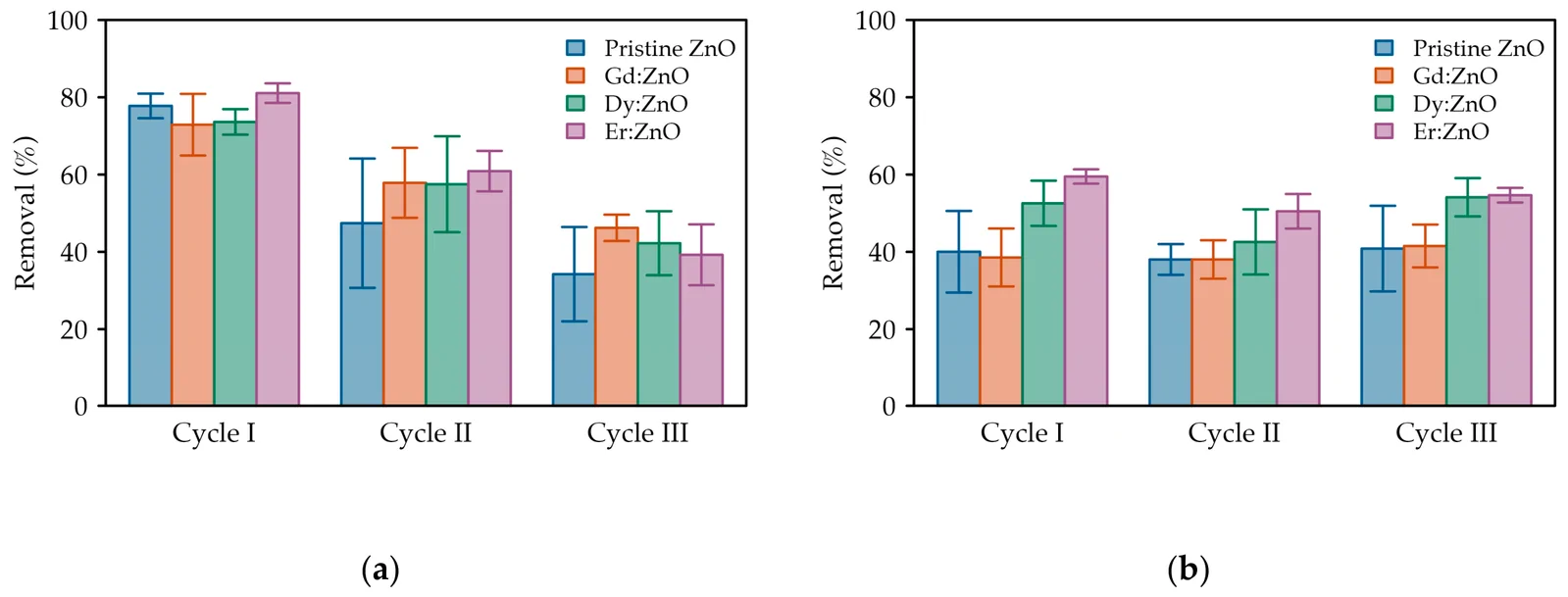
Removal efficiencies obtained using pristine and RE-modified ZnO nanorods during three consecutive treatment cycles at 800 rpm: (**a**) RB5. (**b**) BG. The catalysts were reused without intermediate regeneration. Bars represent mean values from three independent experiments; error bars indicate standard deviations.

The operational reusability of pristine and RE-modified ZnO nanorods was evaluated at 800 rpm, at which the highest removal efficiencies for both dyes were obtained. The catalysts were subjected to three consecutive treatment cycles without intermediate washing or regeneration. After each cycle, the treated solution was discarded and replaced with a fresh dye solution. The removal efficiencies obtained for RB5 and BG are presented in Figure 14a,b, respectively.

For the anionic RB5 (Figure 14a), all four catalysts showed a progressive loss of activity. Er:ZnO exhibited the highest initial removal efficiency of approximately 81%, which decreased to approximately 61% during the second cycle and 39% during the third cycle, corresponding to a relative activity loss of approximately 52%. A comparable decrease from approximately 78% to 34% was observed for pristine ZnO. Gd:ZnO and Dy:ZnO showed somewhat better activity retention, reaching approximately 46% and 42% removal, respectively, during the third cycle. Consequently, all three RE-modified materials provided higher mean removal efficiencies than pristine ZnO during the second and third cycles. The progressive deactivation is consistent with the stronger interaction of RB5 with the catalyst surfaces indicated by the preceding experiments and may result from the accumulation of RB5 or its transformation products at active surface sites.

A different behavior was observed for BG (Figure 14b). The removal efficiencies of pristine ZnO and Gd:ZnO remained nearly constant throughout the three cycles. Dy:ZnO and Er:ZnO showed moderate decreases during the second cycle, followed by partial or complete recovery during the third cycle. No progressive deactivation was therefore observed for BG, and the RE-modified catalysts, particularly Dy:ZnO and Er:ZnO, retained higher removal efficiencies than pristine ZnO after three cycles. The contrasting cycling behavior of RB5 and BG is consistent with their different affinities for the catalyst surfaces: stronger accumulation of RB5-derived species would progressively restrict accessible surface sites, whereas the weaker surface interaction of BG would produce less persistent inhibition.

**Figure 15:**
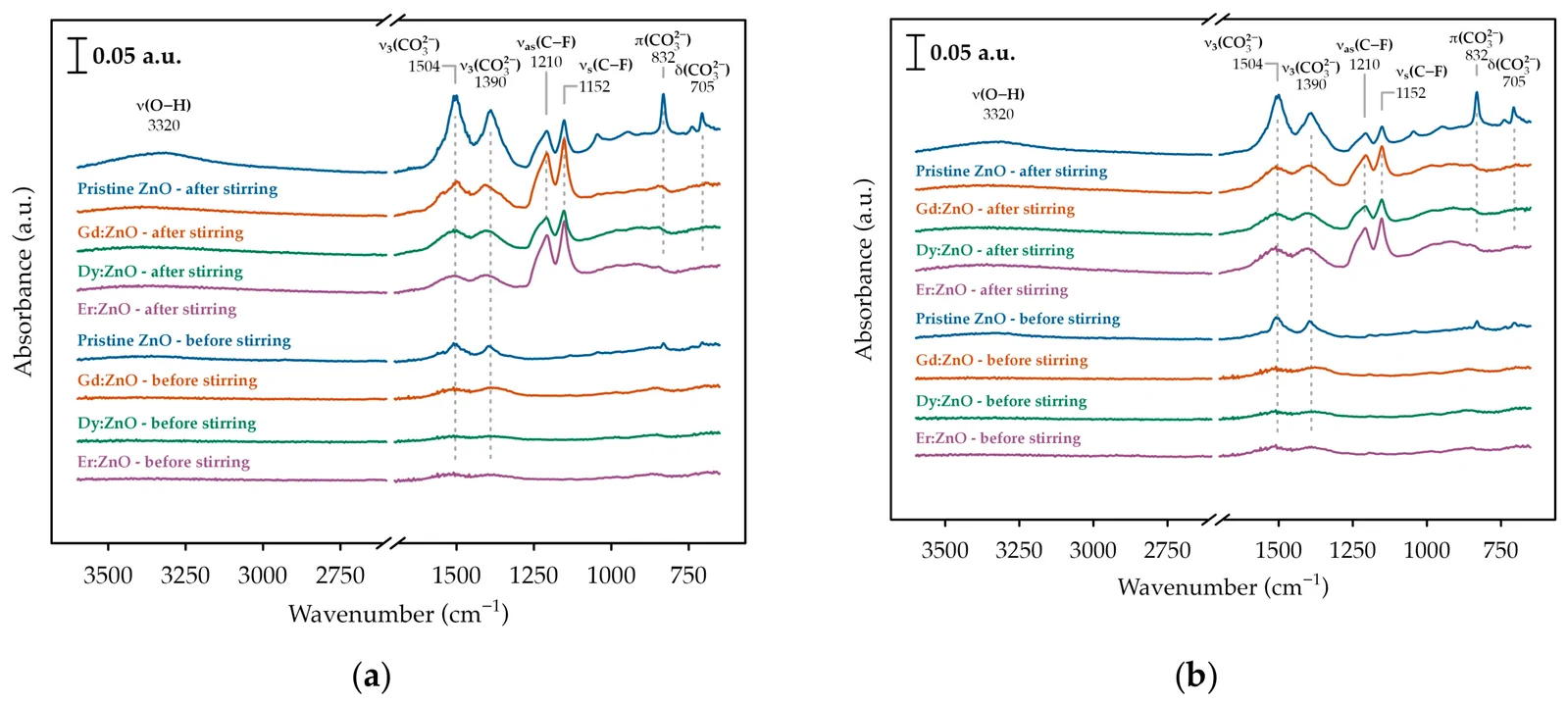
ATR-FTIR spectra of pristine and RE-modified ZnO nanorods after 24 h exposure to dye solutions without stirring and after three consecutive tribocatalytic treatment cycles at 800 rpm: (**a**) RB5. (**b**) BG.

Surface changes accompanying catalyst reuse were examined by ATR-FTIR spectroscopy (Figure 15).

After 24 h exposure to the dye solutions without stirring, the RE-modified ZnO showed only minor changes relative to the as-prepared powders (Figure 7a), whereas pristine ZnO developed carbonate-related bands. These became more pronounced after stirring and reuse, particularly for pristine ZnO. The bands at approximately 1504 and 1390 cm^−1^ are assigned to ν_3_(CO_3_^2−^), while those at 832 and 705 cm^−1^ correspond to π(CO_3_^2−^) and δ(CO_3_^2−^), respectively [46,47,48]. Their development is consistent with the formation of surface zinc carbonate or hydroxycarbonate species, which may restrict accessible active sites and contribute to deactivation [68,69]. These changes, however, are observed both for RB5 (Figure 15a) and BG (Figure 15b), indicating that carbonate formation alone cannot explain the stronger progressive deactivation observed for RB5. No apparent FTIR bands could be attributed to retained dyes or intermediates, possibly because of their low concentrations.

After stirring and reuse, all four catalysts exhibited new bands at 1210 and 1152 cm^−1^, which could be assigned to ν_as_(C-F) and ν_s_(C-F), respectively, and are thus consistent with PTFE [70]. This observation suggests abrasion of the PTFE-coated stirring bar and transfer of PTFE material to the recovered catalysts. Such transfer may also affect surface accessibility and introduces secondary contamination, highlighting the need to account for stirring-bar wear in future tribocatalytic treatment studies.

**Figure 16:**
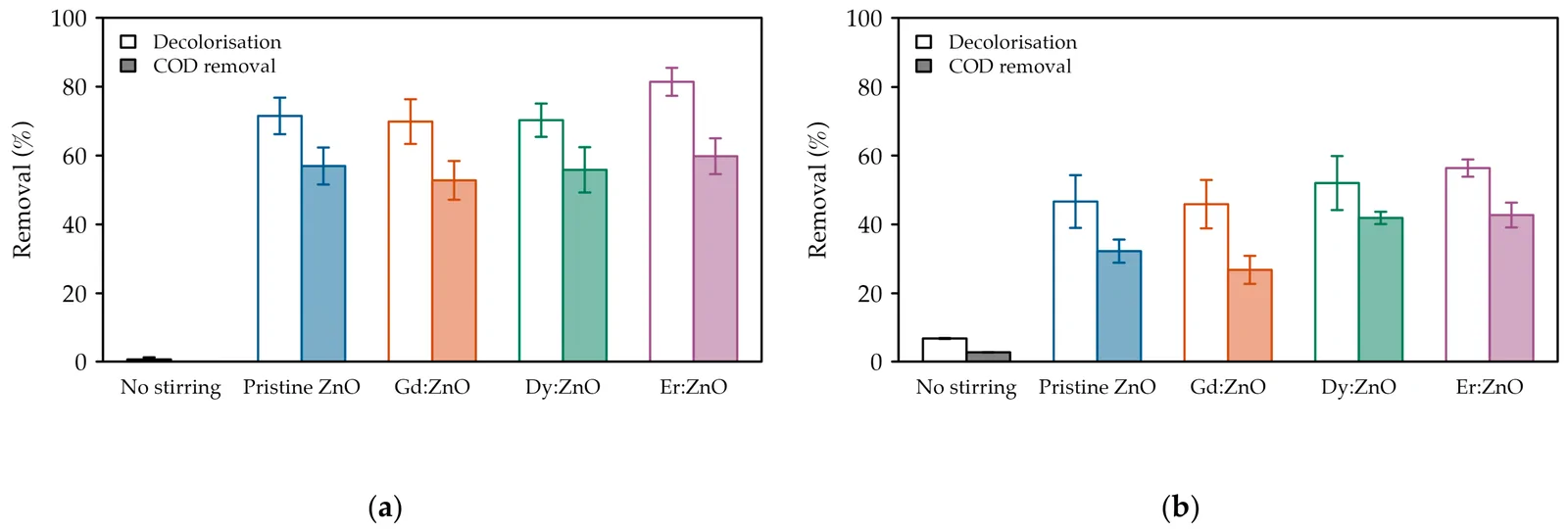
Comparison of decolorization and COD removal after 24 h using pristine and RE-modified ZnO nanorods at 800 rpm with a PTFE-sealed stirring bar: (**a**) RB5. (**b**) BG. Results at 800 rpm represent mean values from three independent experiments. The “No stirring” controls show the mean decolorization and COD removal after adsorption equilibration across all four catalysts, relative to the catalyst-free dye solutions. Error bars indicate standard deviations.

Because dye removal in the preceding experiments was monitored spectrophotometrically, COD measurements were performed to determine whether decolorization was accompanied by a reduction in the oxidizable organic content of the solutions. The decolorization and COD removal efficiencies measured after 24 h are compared in Figure 16.

For RB5 (Figure 16a), decolorization efficiencies of approximately 70–81% were accompanied by COD removal efficiencies of approximately 53–60%. Er:ZnO produced both the highest decolorization and the highest COD removal, whereas comparable COD removal efficiencies were obtained using pristine ZnO, Gd:ZnO, and Dy:ZnO. For BG (Figure 16b), decolorization ranged from approximately 46% to 56%, while COD removal ranged from approximately 27% to 43%. Among the investigated materials, Er:ZnO and Dy:ZnO exhibited the highest COD removal efficiencies for BG, consistent with their higher decolorization efficiencies at 800 rpm.

For both dyes, COD removal was lower than decolorization by approximately 10–21 percentage points. This difference indicates that disruption of the dye chromophores and formation of colorless or weakly absorbing transformation products occurred more readily than further oxidation of these products, as commonly observed during oxidative dye treatment [31]. Nevertheless, the appreciable decrease in COD demonstrates that decolorization was accompanied by a reduction in the oxidizable organic load and is consistent with partial mineralization rather than only loss of color. Before stirring, only minor decreases in absorbance and COD were observed after adsorption equilibration, indicating a limited contribution from adsorption. The corresponding equilibrated values were used as the initial baselines for calculating decolorization and COD removal during the subsequent 24 h treatment.

Table 3 compares the tribocatalytic dye removal obtained in this study with selected literature reports. Although the present catalysts exhibited relatively low average removal rates, their performance was of the same order of magnitude as that reported for ZnO nanorods. Direct comparisons should nevertheless account for differences in the dyes, catalyst loadings, friction pairs, and stirring conditions.

## 4. Conclusions

Pristine ZnO nanorods were successfully obtained through the simple thermal decomposition of zinc acetate dihydrate and subsequently modified with Gd_2_O_3_, Dy_2_O_3_, or Er_2_O_3_ by incipient wetness. The modification introduced surface-associated nanocrystalline rare-earth oxide phases while preserving the rod-like morphology and hexagonal wurtzite structure of the parent ZnO. This preparation route therefore provided a straightforward means of producing structurally comparable pristine and RE-modified ZnO tribocatalysts.

Tribocatalytic activity depended strongly on both the stirring rate and the identity of the dye. The most pronounced activity was observed for the anionic RB5: at 800 rpm, Er:ZnO reached approximately 81% removal after 24 h, with a k_app_ of approximately 0.074 h^−1^. Removal of the cationic BG was lower and less consistently affected by RE modification. Nevertheless, Er:ZnO again showed the highest activity at 800 rpm, reaching approximately 56% removal with a k_app_ of approximately 0.040 h^−1^. Thus, its maximum BG removal was 25 percentage points lower than that obtained for RB5. The contrasting adsorption, scavenger, and reusability results suggested differences in the surface interactions and reaction pathways of the two dyes. The measured pH values remained below the literature-reported isoelectric point of ZnO nanorods, consistent with positively charged surfaces favoring electrostatic attraction of anionic RB5. ATR-FTIR revealed carbonate formation and transfer of PTFE from the stirring bars to the recovered catalysts. Their possible contributions to catalyst deactivation and secondary contamination warrant further investigation. Carbonate formation also highlights the chemical sensitivity of the catalyst surfaces, making possible Zn and RE release relevant to future stability assessments, although metal-ion leaching was outside the scope of the present study. Nevertheless, COD removal of 53–60% for RB5 and 27–43% for BG indicated a reduction in the oxidizable organic load, consistent with partial mineralization.

Overall, the results demonstrate that ZnO nanorods prepared by a simple thermal route possess measurable tribocatalytic activity under mechanical stirring in darkness. RE modification produced some improvements, most notably for Er:ZnO, but its effect was incremental and strongly dependent on the target dye rather than uniformly pronounced. Consequently, the benefits commonly associated with RE modification in photocatalysis cannot be assumed to transfer directly to tribocatalytic systems. Further work should therefore clarify how pollutant charge, molecular structure, surface affinity, and RE-oxide composition govern the observed activity differences. Beyond pollutant removal, accessible ZnO-based materials provide useful platforms for investigating mechanically driven chemical transformations. Improving the understanding of tribocatalytic mechanisms may therefore support their development for other applications, including mechanochemical synthesis.

## Figures and Tables

**Table 1:** ZnO unit-cell parameters (a, b, c, and V) and RE_2_O_3_ phase content obtained by Rietveld refinement, along with crystallite sizes (D_XRD_) and microstrain for pristine and RE-modified ZnO tribocatalysts.

Sample	ZnO Phase					RE_2_O_3_ Phase		R—Values		
	a = b (Å)	c (Å)	V (Å^3^)	D_XRD_ (nm) ^a^	Strain (%) ^a^	D_XRD_ (nm) ^b^	vol. (%)	R_p_	R_wp_	R_exp_
Pristine ZnO	3.2494	5.2047	47.592	62.80	0.235	–	–	11.09	14.67	3.19
Gd:ZnO	3.2495	5.2047	47.595	67.11	0.234	10.57	2.18	9.85	12.91	0.73
Dy:ZnO	3.2500	5.2047	47.609	65.70	0.235	10.12	2.48	9.02	11.68	0.56
Er:ZnO	3.2507	5.2064	47.646	64.71	0.236	10.85	2.45	9.54	12.60	0.59

^a^ Obtained by size-strain analysis of the ZnO (100), (002), (101), (102), (110), and (103) reflections. ^b^ Obtained using the Scherrer equation for the RE_2_O_3_ (222) reflection.

**Table 2:** Surface composition of the pristine and RE-modified ZnO catalysts determined by XPS.

Sample	Zn 2p Zn^2+^ (at. %)	O 1s Components (at. %)			C 1s Components (at. %)		Gd 3d Gd^3+^ (at. %)	Dy 4d Dy^3+^ (at. %)	Er 4d Er^3+^ (at. %)
		Lattice O^2−^	Surface –OH	Adsorbed Species	C–C, C–H	CO_3_^2−^			
Pristine ZnO	48.32	21.76	8.51	7.40	14.00	–	–	–	–
Gd:ZnO	31.80	17.68	10.80	11.26	21.41	4.72	2.34	–	–
Dy:ZnO	41.87	21.81	10.48	8.56	12.99	3.23	–	1.06	–
Er:ZnO	41.43	21.68	9.18	8.07	13.99	3.12	–	–	2.54

**Table 3:** Comparison of tribocatalytic dye removal in the present study and selected literature reports.

Catalyst	Reaction Conditions	Friction Pair	Speed	Removal/Time	Average Removal * (mg g^−1^ h^−1^)	Ref.
Ba_0.75_Sr_0.25_TiO_3_ nanoparticles	5 ppm RhB; 1 mg mL^−1^ catalyst	1 × PTFE bar on glass	300 rpm	99% in 12 h	≈0.41	[15]
		4 × PTFE bar on glass		99% in 3 h	≈1.65	
ZnO nanorods	5 ppm RhB; 1 mg mL^−1^ catalyst	1 × PTFE bar on glass	1000 rpm	99.8% in 60 h	≈0.08	[16]
ZnO nanostars	5 ppm RhB; 2 mg mL^−1^ catalyst	1 × PTFE bar on glass	600 rpm	95% in 12 h	≈0.20	[17]
Si powder	10 ppm RhB; 3.33 mg mL^−1^ catalyst	PTFE disk on glass	600 rpm	98.7% in 3.5 h	≈0.85	[20]
Biochar-ZnO composite	5 ppm RhB; 2 mg mL^−1^ catalyst	1 × PTFE bar on quartz	450 rpm	72% in 1.25 h	≈1.44	[21]
		1 × PTFE bar on polyimide		90% in 1.25 h	≈1.80	
ZnO nanoparticles	5 ppm RhB; 10 mg mL^−1^ catalyst	PTFE disk on glass	400 rpm	99% in 1.5 h	≈0.33	[23]
Er_2_O_3_ nanoparticles	5 ppm RhB; 0.83 mg mL^−1^ catalyst	1 × PTFE bar on roughened glass	900 rpm	97.54% in 5 h	≈1.17	[63]
Er:ZnO nanorods	10 ppm RB5/BG; 5 mg mL^−1^ catalyst	1 × PTFE bar on glass	800 rpm	81% (RB5) in 24 h	≈0.07	This work
				56% (BG) in 24 h	≈0.05	

* Recalculated based on available information in the respective manuscripts.

## Data Availability

The original contributions presented in this study are included in the article. Further inquiries can be directed to the corresponding author.
